# Isolated Infrarenal Aortic Dissection Presenting With Predominant Neurological Symptoms Without Limb Ischemia: A Case Report

**DOI:** 10.7759/cureus.106604

**Published:** 2026-04-07

**Authors:** Nabil Al_Madhwahi, Ali Fadhel, Al-Ezy M Hamoud, Yahya A Al-Modwahi, Emad A Halboob

**Affiliations:** 1 Department of Vascular Surgery, Faculty of Medicine and Health Science, Sana’a University, Sana'a, YEM; 2 Department of Cardiac Surgery, Al-Thawra Modern General Hospital, Sana’a, YEM; 3 Department of Vascular Surgery, Al-Thawra Modern General Hospital, Sana'a, YEM

**Keywords:** aortobifemoral bypass, atypical presentation, infrarenal aortic dissection, isolated abdominal aortic dissection, lower limb weakness, neurological symptoms

## Abstract

Isolated abdominal aortic dissection (IAAD) is a rare clinical entity, particularly when confined to the infrarenal segment. The presentation is often atypical, leading to delayed diagnosis. We present the case of a 60-year-old female with a history of hypertension who presented to the emergency department with a five-day history of right lower limb pain and inability to stand. Her symptoms began as a sudden, severe, electric shock-like pain in the right flank radiating down the right leg, which subsided after 15 minutes and was replaced by persistent numbness and generalized weakness. Physical examination revealed normal lower limb coloration, palpable pulses, and no signs of acute limb ischemia, lacking the classic five Ps. Echocardiography was unremarkable for proximal aortic pathology. Abdominal CT angiography (CTA) revealed an isolated infrarenal aortic dissection extending to the aortic bifurcation and terminating in the right iliac arteries, with a completely thrombosed false lumen. Given the persistent symptoms suggestive of malperfusion and the absence of suitable endovascular landing zones, the patient underwent an open aortobifemoral bypass using a polytetrafluoroethylene graft. Postoperatively, her neurological symptoms resolved completely, and she was discharged on the 10thpostoperative day without complications. This case highlights the importance of considering aortic dissection in patients presenting with unexplained neurological symptoms, even in the absence of overt limb ischemia. Early diagnosis with CTA and appropriate surgical intervention can lead to excellent outcomes.

## Introduction

Aortic dissection is a life-threatening cardiovascular emergency that typically involves the thoracic aorta. Isolated abdominal aortic dissection (IAAD) is an extremely rare condition, accounting for approximately 1-4% of all aortic dissections, depending on the classification used [1,2]. Unlike thoracic aortic dissections, which are well classified by the Stanford and DeBakey systems, IAAD lacks a standardized classification, and its natural history remains poorly understood [3]. The most common risk factor for IAAD is hypertension, which has been identified in the majority of reported cases, followed by atherosclerosis, hyperlipidemia, and smoking [1,4]. The pathophysiology of IAAD involves an intimal tear that allows blood to enter the medial layer, creating a false lumen that may compromise branch vessel perfusion. Infrarenal involvement is particularly uncommon, likely due to lower hemodynamic stress compared to the thoracic aorta.

The classic presentation of aortic dissection includes sudden, severe tearing chest, abdominal, or back pain. However, atypical presentations, such as isolated neurological deficits, can occur and often lead to misdiagnosis or delayed treatment [5,6]. Neurological manifestations, including lower limb weakness, paraplegia, or paresthesia, are usually attributed to spinal cord ischemia caused by the occlusion of radicular arteries, particularly the artery of Adamkiewicz, originating from the dissected aorta [7]. Such presentations may occur even in the absence of overt limb ischemia, posing a diagnostic challenge.

Lower limb neurological symptoms, including weakness and numbness, are commonly attributed to a wide range of etiologies. These include spinal pathologies such as intervertebral disc herniation and spinal cord ischemia, peripheral neuropathies, and, less commonly, vascular causes leading to impaired perfusion of neural structures. Differentiating between these causes can be challenging, particularly in the absence of classical vascular signs, and may lead to delayed or missed diagnoses.

We present a rare case of an isolated infrarenal aortic dissection in a 60-year-old female who presented predominantly with neurological symptoms, namely lower limb weakness and numbness, which represent an atypical manifestation of IAAD, in the absence of the classic signs of acute limb ischemia. This report discusses the diagnostic challenges, the rationale for open surgical management, and the favorable outcome of this unusual presentation.

## Case presentation

A 60-year-old female patient presented to the emergency department with a five-day history of right lower limb pain and progressive inability to stand. Her medical history was significant for hypertension, which had been managed with regular medication for the past three months prior to presentation. She had no other past medical or surgical history.

The patient described a sudden onset of severe, electric shock-like pain originating in her right flank, shooting down her right leg, and radiating distally. This acute pain lasted for approximately 15 minutes before subsiding spontaneously. However, the pain was immediately replaced by persistent numbness and significant generalized weakness, rendering her unable to stand on both lower limbs. She denied any frank paralysis but emphasized the profound weakness. There were no associated symptoms such as fever, chest pain, abdominal pain, or trauma.

On physical examination, the patient was conscious, oriented, cooperative, and hemodynamically stable (blood pressure: 150/90 mmHg, heart rate: 88 bpm). She was alert and oriented. Cardiovascular and respiratory examinations were unremarkable.

Crucially, the examination of both lower limbs showed normal coloration, no hair loss, no dilated veins, no muscle atrophy, no swelling, no ulceration, and no gangrene. The capillary refill time was normal, and there was no tenderness or tense compartment. Distal pulses were palpable bilaterally. Notably, she lacked all the classic five Ps (pain, pallor, pulselessness, paresthesia, and paralysis) of acute limb ischemia.

Neurological examination revealed symmetrical reduction in motor strength (Medical Research Council grade 4/5) affecting both lower limbs across all major muscle groups, without focal myotomal distribution. Deep tendon reflexes were preserved and symmetrical. Sensory examination demonstrated subjective paresthesia without a defined dermatomal pattern or sensory level. There was no evidence of saddle anesthesia. Bowel and bladder functions were intact, with preserved urinary continence. Anal tone was normal, and the bulbocavernosus reflex was intact. These findings made acute compressive neurological conditions such as cauda equina syndrome unlikely.

Initial differential diagnoses included spinal pathology, peripheral neuropathy, and vascular etiologies. To investigate the cause of her symptoms, transthoracic echocardiography was performed, which showed no evidence of proximal aortic involvement or a cardiac source of emboli.

**Figure 1:**
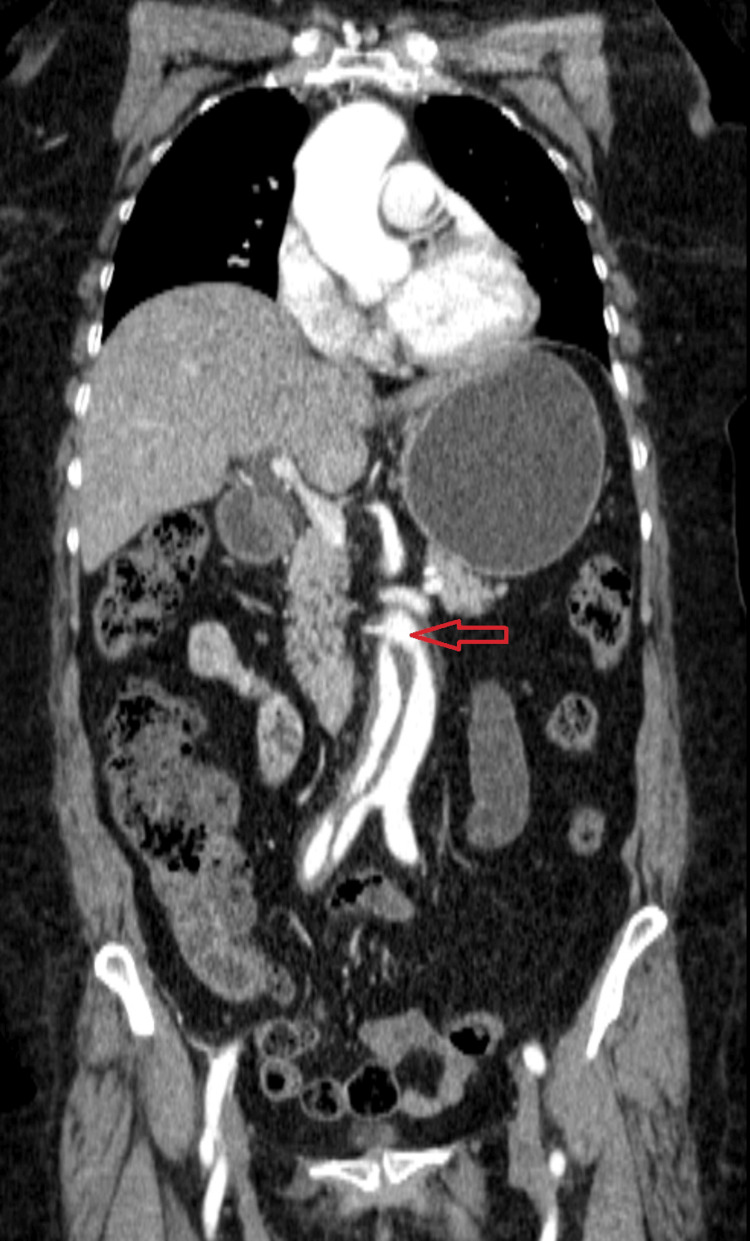
Coronal contrast-enhanced CTA demonstrating an infrarenal abdominal aortic dissection The image shows an intimal flap (red arrow) within the infrarenal aorta, just distal to the renal arteries, resulting in a double-lumen appearance characteristic of aortic dissection and confirming the infrarenal origin of the lesion. CTA, CT angiography

Subsequently, an abdominal CT angiography (CTA) was obtained. The CTA demonstrated an isolated infrarenal aortic dissection (Figure 1).

**Figure 2:**
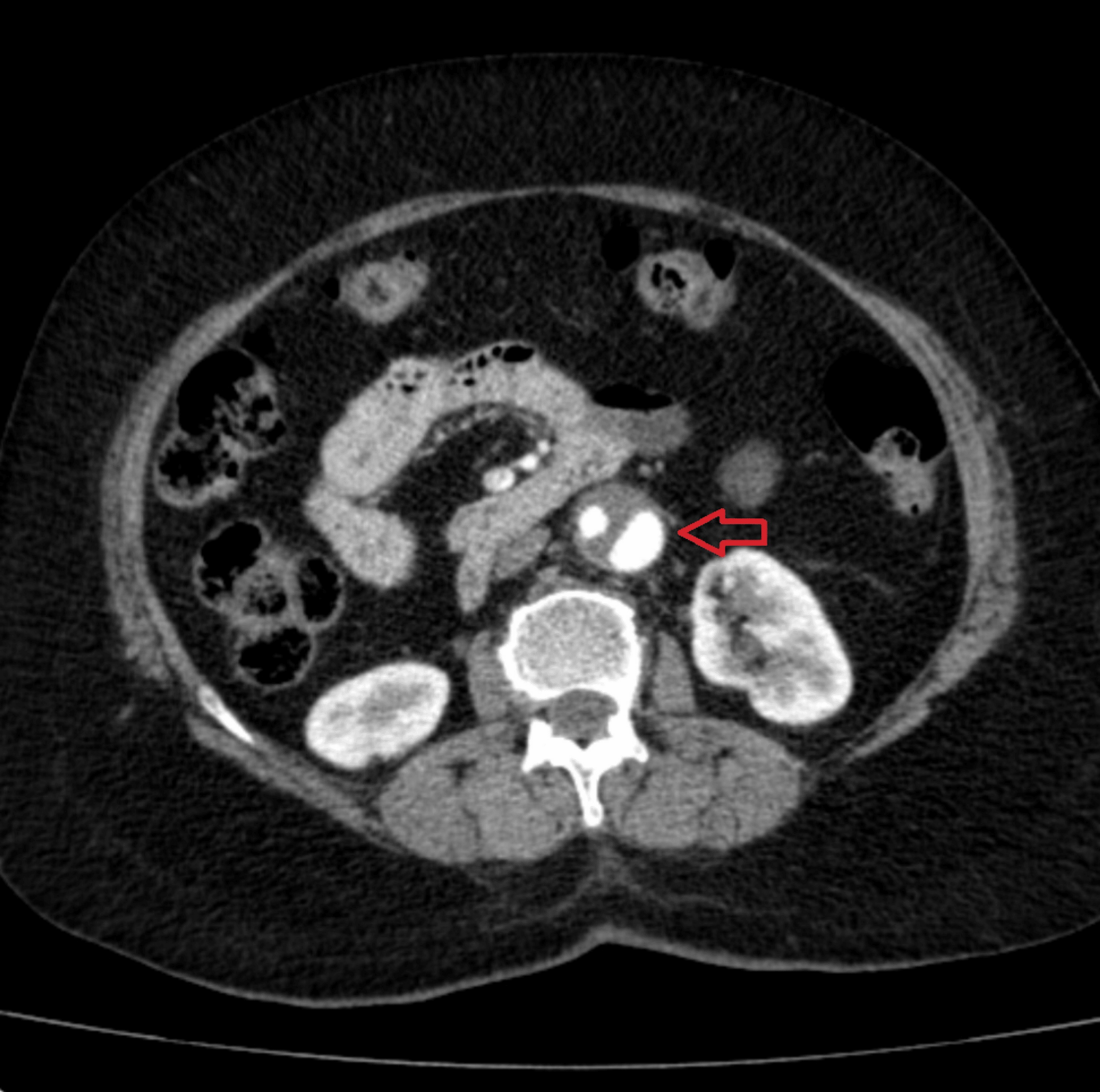
Axial contrast-enhanced CTA demonstrating an infrarenal abdominal aortic dissection The axial image demonstrates an intimal flap (red arrow) within the infrarenal aorta, resulting in a double-lumen appearance characteristic of aortic dissection, with clear differentiation between the true and false lumens. CTA, CT angiography

**Figure 3:**
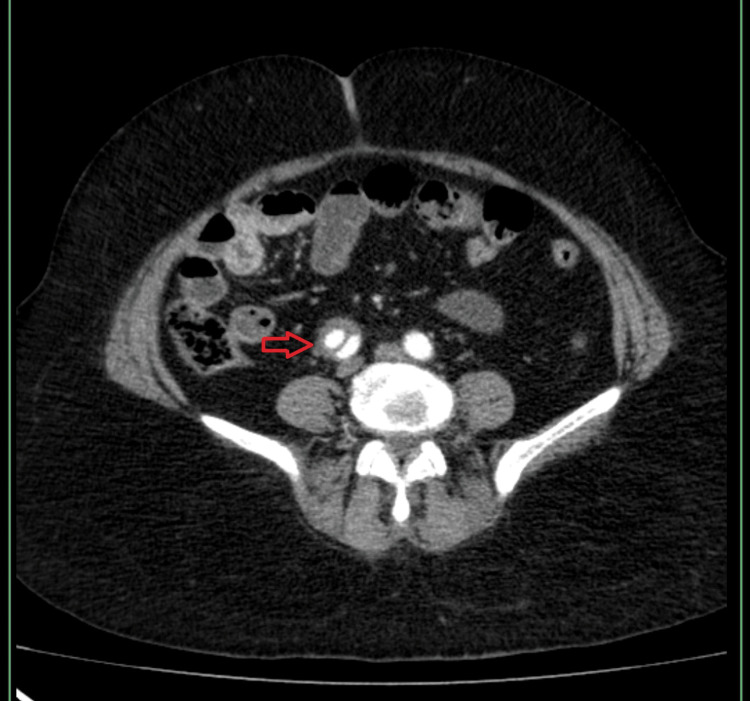
Axial contrast-enhanced CTA demonstrating distal extension of the infrarenal aortic dissection The image shows a persistent intimal flap (red arrow) within the distal infrarenal aorta, maintaining a double-lumen appearance and confirming distal propagation of the dissection toward the iliac arteries. CTA, CT angiography

The dissection flap originated immediately distal to the renal arteries, confirming its infrarenal nature, and extended down to the aortic bifurcation (Figure 2), terminating distally in the right common iliac and right external iliac arteries (Figure 3).

**Figure 4:**
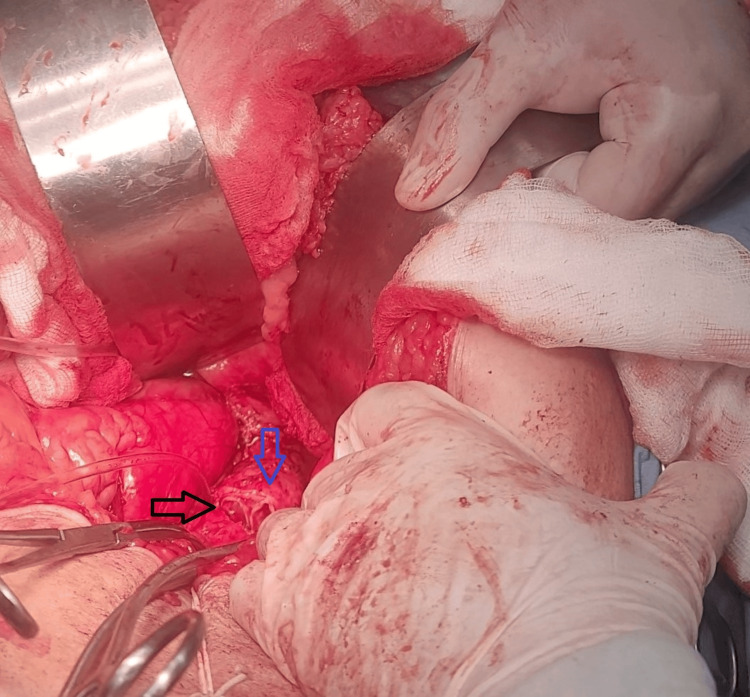
Intraoperative photograph demonstrating the infrarenal aortic dissection following aortotomy The image shows the exposed infrarenal aorta during open surgical repair. An intimal flap (blue arrow) is directly visualized, separating the true and false lumens, with intraluminal thrombus (black arrow) within the false lumen, confirming complete thrombosis.

The true lumen remained patent, while the false lumen was completely thrombosed (Figure 4). No visceral branch involvement or aneurysmal dilatation was identified.

Upon establishing the diagnosis, the patient was immediately started on medical management, which included anticoagulation and blood pressure control. Given the persistence of neurological symptoms suggestive of malperfusion and the anatomical extent of the dissection, intervention was indicated. Open surgical repair was preferred over endovascular treatment due to the involvement of the iliac arteries and the absence of suitable endovascular landing zones.

**Figure 5:**
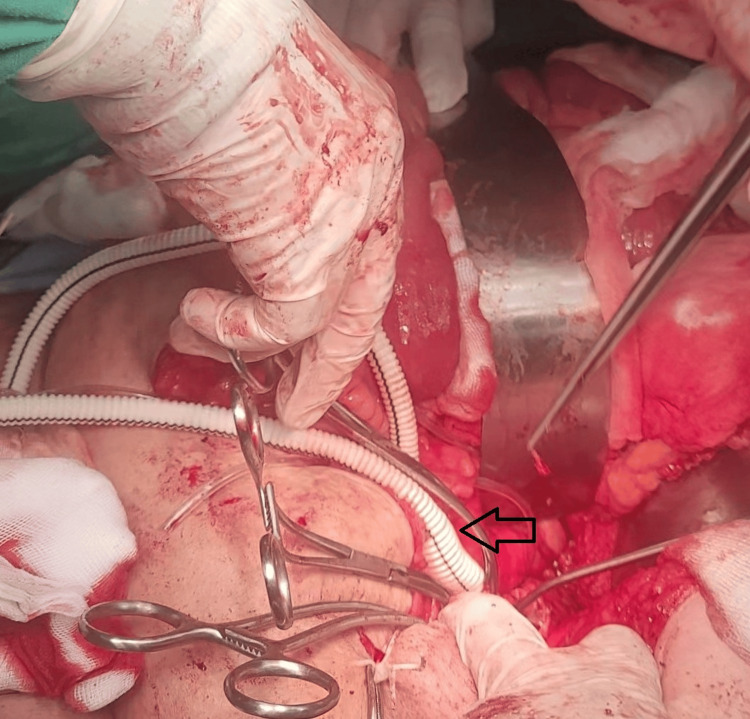
Intraoperative photograph demonstrating aortobifemoral bypass graft placement The image shows a bifurcated PTFE graft (black arrow) following proximal anastomosis to the infrarenal aorta, with the graft limbs directed toward the femoral arteries for lower limb revascularization. PTFE, polytetrafluoroethylene

The patient underwent an open aortobifemoral bypass using a polytetrafluoroethylene (PTFE) synthetic graft (Figure 5).

The procedure was performed via a midline laparotomy. After achieving proximal and distal control of the aorta, a complete transection of the aorta was performed just distal to the renal vessels. Upon aortotomy, two distinct lumens were clearly identified: the true lumen and the false lumen, which was filled with thrombus (Figure 4). The thrombus within the false lumen was evacuated. A small segment of the graft material was used to obliterate the false lumen and reinforce the aortic wall using a polypropylene suture (Prolene 5-0; Ethicon, Inc., Raritan, NJ, USA).

**Figure 6:**
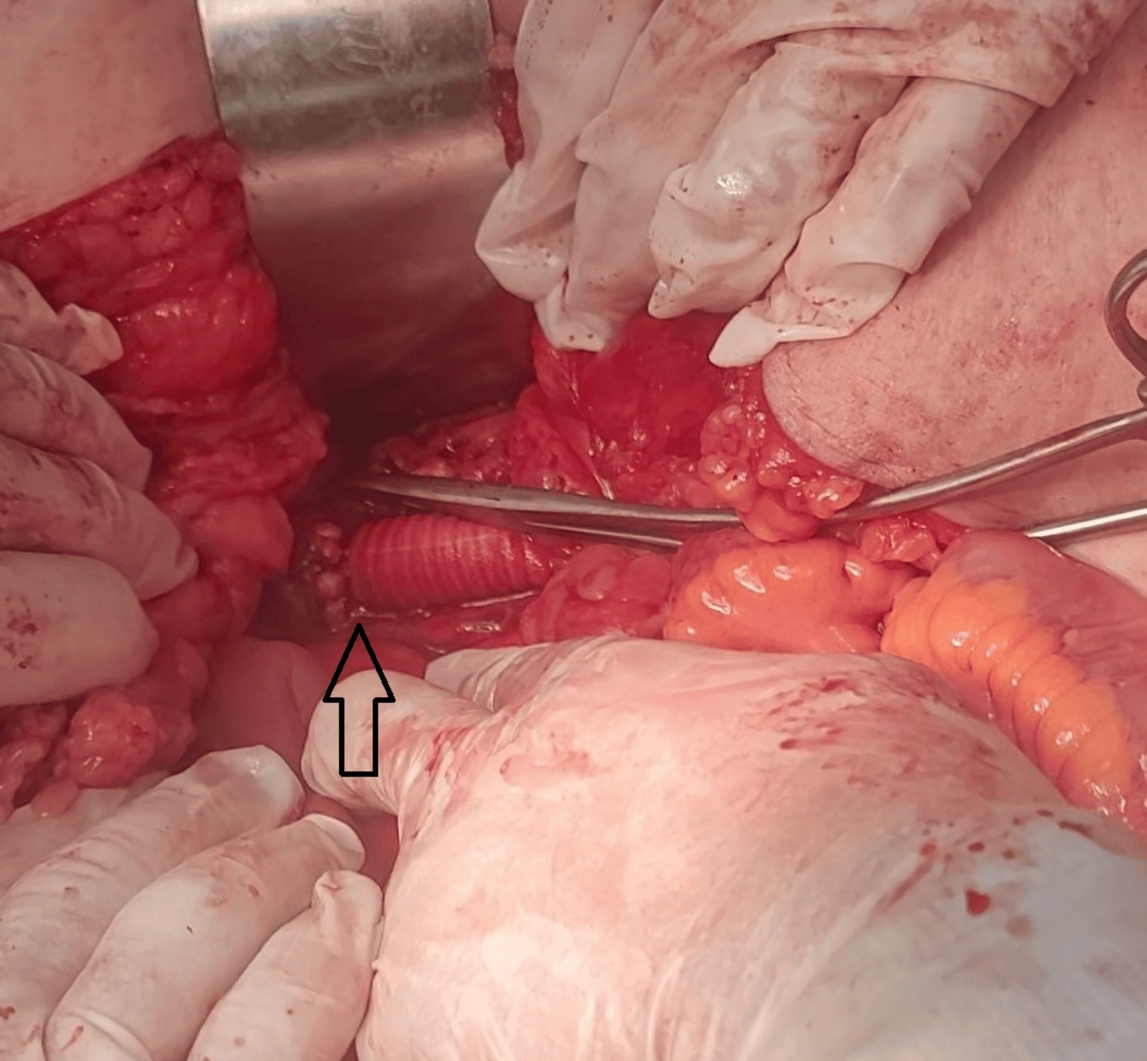
Intraoperative photograph demonstrating the completed aortobifemoral bypass reconstruction The image shows the implanted bifurcated PTFE graft (black arrow) following completion of the vascular reconstruction. The graft limb is appropriately positioned toward the distal vessels, restoring aortic continuity and excluding the dissected segment. PTFE, polytetrafluoroethylene

An end-to-end aorto-graft anastomosis was then performed proximally using a polypropylene suture (Prolene 5-0; Ethicon, Inc.) (Figure 6).

Distally, end-to-side anastomoses were performed to both common femoral arteries using a polypropylene suture (Prolene 6-0; Ethicon, Inc.). Drains were placed in the pelvis, and vacuum drains were installed in both groins. After ensuring good hemostasis, all incisions were closed in layers using polyglactin 910 (Vicryl; Ethicon, Inc.) for the fascia and subcutaneous tissue and a stapler for the skin. The patient did not experience any serious complications during the surgery and was transferred to the ICU for close monitoring.

Postoperatively, the patient had an uneventful recovery. She was managed with continued anticoagulation, antihypertensive medications, broad-spectrum antibiotics, proton pump inhibitors, and analgesics. She remained in the ICU for three days. A follow-up Doppler ultrasound demonstrated satisfactory graft patency with adequate distal flow. Following the removal of the drains, she was transferred to the regular ward. Her numbness and inability to stand resolved completely, and she regained full ambulation. The patient was discharged on the tenth postoperative day without any complications. Upon discharge, a comprehensive follow-up schedule was established, including a planned CTA to closely monitor her aortic anatomy and ensure the long-term durability of the repair.

## Discussion

IAAD is a rare vascular pathology that has garnered increasing attention in recent literature. According to the systematic review and meta-analysis by Wu et al., IAAD accounts for only 1.7% of all aortic dissections, with the infrarenal aorta being the most commonly affected segment [1]. A subsequent meta-analysis by Liu et al. corroborated these findings, reporting that IAADs are predominantly spontaneous and infrarenal, with approximately half being acute and symptomatic [2]. The etiology of IAAD is primarily associated with hypertension, which is present in the majority of cases, as seen in our patient [4]. Other risk factors include atherosclerosis, smoking, hyperlipidemia, and connective tissue disorders [1,2]. The pathophysiology involves an intimal tear that allows blood to enter the media, creating a false lumen that may remain patent, partially thrombose, or completely thrombose.

In our case, the false lumen was completely thrombosed, a finding that carries important prognostic implications. A meta-analysis by Zhang et al. demonstrated that complete thrombosis of the false lumen is associated with improved long-term survival in patients with aortic dissection compared to a patent or partially thrombosed false lumen [8]. This favorable false lumen status may have contributed to the absence of frank limb ischemia in our patient despite the extensive dissection.

The clinical presentation of IAAD is highly variable. While most patients present with acute abdominal or back pain, atypical presentations can occur and pose significant diagnostic challenges. Our patient presented with transient electric shock-like pain followed by persistent lower limb weakness and numbness, a presentation that could easily be mistaken for a neurological or musculoskeletal condition.

Alternative diagnoses, including spinal pathology and peripheral neuropathy, were carefully considered. However, the absence of focal neurological deficits, preserved reflexes, normal sphincter function, and confirmatory findings on CTA made these diagnoses unlikely. These findings suggest that transient hypoperfusion rather than fixed arterial occlusion may explain the neurological presentation.

Neurological symptoms in aortic dissection are relatively rare and are typically related to compromised spinal cord perfusion due to the involvement of segmental arteries supplying the spinal cord [7]. However, in the absence of dedicated spinal imaging, the exact mechanism remains speculative. Sakurai et al. recently reported a similar case of painless aortic dissection presenting with transient bilateral lower limb weakness, emphasizing that neurological deficits may be the sole presenting feature of aortic dissection [5].

In this case, the absence of classical signs of limb ischemia despite significant neurological symptoms highlights the complexity of presentation. Preserved distal perfusion suggests that symptoms may have been related to transient hypoperfusion or nerve root compression rather than fixed arterial occlusion.

CTA is the diagnostic modality of choice for IAAD, providing high-resolution visualization of the dissection, its extent, and branch vessel involvement [9]. In our case, CTA accurately delineated the infrarenal origin of the dissection and its extension into the right iliac arteries, guiding the surgical approach. The importance of CTA in the diagnostic workup of patients with atypical presentations cannot be overstated, as it remains the most reliable modality for confirming or excluding aortic dissection [9].

The management of IAAD remains controversial due to the lack of large randomized controlled trials. Treatment options include conservative medical management, endovascular aneurysm repair (EVAR), and open surgical repair [10]. Conservative management with strict blood pressure control and anticoagulation is often reserved for asymptomatic patients with uncomplicated dissections. However, a recent prospective cohort study by Wu et al. demonstrated that conservative treatment is associated with significantly worse survival compared to invasive interventions (p = 0.043) and recommended a more aggressive treatment approach, particularly for symptomatic patients with a patent false lumen [3]. The meta-analysis by Liu et al. reported a 30-day all-cause mortality of 3% and a long-term mortality of 8% for the overall IAAD population, with re-intervention rates of 18% in conservatively managed patients [2]. These data support the rationale for surgical intervention in our symptomatic patient.

Indications for surgical or endovascular intervention in IAAD include persistent pain, rapid aortic expansion, impending rupture, and malperfusion syndromes, such as the neurological deficits observed in our patient [10,11]. While EVAR has become increasingly popular due to its lower perioperative morbidity, open surgical repair remains a durable and effective option, particularly in cases with complex anatomy or unsuitable endovascular access [11,12]. In our case, open surgical repair was preferred over endovascular treatment due to the involvement of the iliac arteries and the absence of suitable endovascular landing zones. The surgical technique involved obliterating the false lumen and reinforcing the aortic wall, which successfully restored normal perfusion and resolved the patient’s neurological symptoms. The complete recovery of her ability to walk underscores the importance of prompt surgical intervention in reversing ischemia-induced neurological deficits.

Several unique aspects of our case merit emphasis. First, the presentation with predominantly neurological symptoms in the absence of the classic five Ps of acute limb ischemia is exceedingly rare in infrarenal aortic dissection and highlights the importance of maintaining a broad differential diagnosis. Second, the complete thrombosis of the false lumen, while potentially protective against acute limb ischemia, did not prevent the development of neurological symptoms, suggesting alternative mechanisms such as transient spinal cord ischemia. Third, the successful outcome following open surgical repair with an aortobifemoral bypass demonstrates the efficacy of this approach in managing symptomatic IAAD, especially when anatomical constraints preclude endovascular options.

## Conclusions

Isolated infrarenal aortic dissection is a rare condition that may present with neurological symptoms without overt limb ischemia. This case highlights the importance of maintaining a high index of suspicion, considering a broad differential diagnosis, and utilizing CTA for accurate diagnosis in patients with unexplained neurological deficits. While the optimal management strategy for IAAD continues to be debated, prompt open surgical intervention, such as an aortobifemoral bypass, may be an effective treatment option in symptomatic patients with unsuitable endovascular anatomy and may result in favorable clinical outcomes.
